# Using Mobile Sensing to Test Clinical Models of Depression, Social Anxiety, State Affect, and Social Isolation Among College Students

**DOI:** 10.2196/jmir.6820

**Published:** 2017-03-03

**Authors:** Philip I Chow, Karl Fua, Yu Huang, Wesley Bonelli, Haoyi Xiong, Laura E Barnes, Bethany A Teachman

**Affiliations:** ^1^ Department of Psychology University of Virginia Charlottesville, VA United States; ^2^ School of Engineering and Applied Science University of Virginia Charlottesville, VA United States; ^3^ Department of Computer Science Missouri University of Science and Technology Rolla, MO United States

**Keywords:** mental health, depression, social anxiety, affect, homestay, mobile health, mHealth

## Abstract

**Background:**

Research in psychology demonstrates a strong link between state affect (moment-to-moment experiences of positive or negative emotionality) and trait affect (eg, relatively enduring depression and social anxiety symptoms), and a tendency to withdraw (eg, spending time at home). However, existing work is based almost exclusively on static, self-reported descriptions of emotions and behavior that limit generalizability. Despite adoption of increasingly sophisticated research designs and technology (eg, mobile sensing using a global positioning system [GPS]), little research has integrated these seemingly disparate forms of data to improve understanding of how emotional experiences in everyday life are associated with time spent at home, and whether this is influenced by depression or social anxiety symptoms.

**Objective:**

We hypothesized that more time spent at home would be associated with more negative and less positive affect.

**Methods:**

We recruited 72 undergraduate participants from a southeast university in the United States. We assessed depression and social anxiety symptoms using self-report instruments at baseline. An app (Sensus) installed on participants’ personal mobile phones repeatedly collected in situ self-reported state affect and GPS location data for up to 2 weeks. Time spent at home was a proxy for social isolation.

**Results:**

We tested separate models examining the relations between state affect and time spent at home, with levels of depression and social anxiety as moderators. Models differed only in the temporal links examined. One model focused on associations between changes in affect and time spent at home within short, 4-hour time windows. The other 3 models focused on associations between mean-level affect within a day and time spent at home (1) the *same* day, (2) the *following* day, and (3) the *previous* day. Overall, we obtained many of the expected main effects (although there were some null effects), in which higher social anxiety was associated with more time or greater likelihood of spending time at home, and more negative or less positive affect was linked to longer homestay. Interactions indicated that, among individuals higher in social anxiety, higher negative affect and lower positive affect within a day was associated with greater likelihood of spending time at home the following day.

**Conclusions:**

Results demonstrate the feasibility and utility of modeling the relationship between affect and homestay using fine-grained GPS data. Although these findings must be replicated in a larger study and with clinical samples, they suggest that integrating repeated state affect assessments in situ with continuous GPS data can increase understanding of how actual homestay is related to affect in everyday life and to symptoms of anxiety and depression.

## Introduction

Our emotional or affective experiences are a central determinant of our behavior, and, in turn, how we behave has an enormous impact on how we feel. Survey results from 2014 showed that, at the trait or more enduring symptom level, 27% of US college students reported feeling “too depressed to function” and 40% reported feeling overwhelming anxiety at least once [1]. Importantly, even subthreshold levels of depression and social anxiety can have a damaging effect on current and future academic performance, relationship quality, and self-esteem [2,3]. Depression and social anxiety are marked by high trait negative affect (ie, the propensity to experience distressing emotions) and low trait positive affect (ie, the propensity to not experience pleasurable emotions) [4]. Not surprisingly, individuals higher in trait negative affect and lower in trait positive affect tend to experience more negative and less positive emotional states (ie, transient fluctuations in mood), as found in past research on depression and social anxiety [5,6].

One key corollary of both trait and state affect is thought to be the degree to which someone seeks out or avoids social contact and engages in activities that provide reinforcement from the environment. To test this idea, researchers typically use questionnaires and ask people to retrospectively report on how positive or negative they have felt and how much social contact they have had over a predetermined period (eg, the last 2 weeks) or in general. Findings generally suggest that positive affect is associated with self-reported approach behavior, such as healthy social engagement, whereas negative affect is associated with self-reported avoidance behavior, such as social isolation [7,8]. While these findings are consistent with the prediction that engaging in activities is associated with healthier emotional functioning, this methodology is limited in its ability to inform researchers about people’s actual experiences in everyday life, such as the ways in which mood and time spent at home (a measure suggestive of social isolation) are linked. One concern is that a wealth of research has documented limitations in people’s ability to retrospectively report on complex behavioral patterns [9]. Yet knowing how affect, symptoms, and time spent at home are actually linked in real time is critical, both to test influential theories and to guide treatment for individuals with dysregulated trait affect, such as persons with anxiety and depressive disorders. For instance, behavioral activation treatment seeks to decrease depressive symptoms by having patients seek out activities that provide reinforcement [10]. To improve behavioral activation and related treatments that strive to reduce withdrawal and avoidance behaviors (as is standard in cognitive behavior therapy for mood and anxiety disorders) [11], we need a more fine-grained understanding of how time spent at home is related to affective experiences in everyday life. Our research can help provide this understanding by using a new approach to integrate passively sensed global positioning system (GPS) data with in situ state affect assessments.

Until recently, mental health researchers had to rely mostly on static and imprecise self-report measures to infer crucial behavioral patterns, such as avoidance and social isolation (unless they were able to invest enormous resources to do in-person observations, which are rare and typically provide only a small sample of a person’s behaviors). Advances in mobile phone technology now make it possible to continuously and unobtrusively monitor where someone is without needing to ask. For example, previous research has found that passively sensed location information can predict depressive symptoms with impressive accuracy [12,13], and researchers have begun to explore passively and actively sensed indicators of stress and health behaviors in college students [14], although little work has focused on how to integrate passively sensed data with affective experiences generated from in situ repeated assessments.

We hypothesized that higher levels of negative affect and lower levels of positive affect (as reflected in trait affect scores from depression and social anxiety symptoms, as well as state affect scores from ecological momentary assessment) would be associated with increased time spent at home. We focused on time spent at home to index isolation because “homestay” is widely recognized by clinicians as an indicator of social disengagement, particularly on college campuses where opportunities for social interaction are plentiful. Moreover, previous research has demonstrated that homestay is a positive predictor of depressive symptoms [13]. Although we had a central hypothesis that time spent at home would be associated with feeling worse (more symptoms, and more negative or less positive state affect), given the novelty of integrating fine-grained location data from GPS sensors with repeated in situ sampling of affect, we did not have specific hypotheses regarding the time course of the homestay-affect links, and therefore explored different models that varied the temporal connections examined. Thus, in addition to testing whether existing theoretical relationships between (trait and state) affect and social isolation could be found in a temporally rich dataset, another contribution of this research is to better understand how best to leverage time-rich data through different models (eg, by looking at mean affect and change in affect as factors associated with concurrent homestay, as well as associations between mean affect, and prior and future homestay).

## Methods

### Participants

In total, 72 undergraduates between 18 and 23 years old (mean 19.8, SD 2.4, 37 female) completed the study in exchange for course credit or payment. Because this research used a novel, customized mobile phone app that was only compatible with Android platforms, we recruited participants with Android phones through email advertisements sent to a university email listserv for undergraduate students, as well as through an undergraduate study participant pool. The sample reported their race/ethnicity as 42% (30/72) white, 38% (27/72) Asian, 4% (3/72) black, 4% (3/72) Latino, and 13% (9/72) multiracial or unspecified. Due to software bugs and compatibility issues that resulted in some participants having little to no data, we excluded 9 participants from data analysis, leaving 63 participants. The study was approved by the local university institutional review board.

### Self-Report Measures

#### Social Anxiety

The Social Interaction Anxiety Scale (SIAS) [15] assesses distress from social interactions and was administered at a baseline laboratory session. Participants rated the degree to which they agreed with 20 statements (eg, “I have difficulty talking with other people”) from 0 (“not at all characteristic of me”) to 4 (“extremely characteristic of me”). The SIAS has been found to differentiate individuals with social anxiety disorder from healthy control participants [15,16]. We examined SIAS scores on a continuous scale (sample mean 29.9, SD 9.6) to obtain the full range of scores (to assess moderation) and in keeping with recommendations from US National Institute of Mental Health Research Domain Criteria: Interim Guidance (Notice Number NOT-MH-11-005) [17]. Based on documented scores among individuals with a diagnosis of social anxiety disorder (mean 40.0, SD 16.0) [15], approximately 16% of our study’s sample likely scored above the mean of a diagnosed sample. Internal consistency of the SIAS in our sample was good (alpha=.83).

#### Depression

We used the 7-item depression subscale of the Depression, Anxiety and Stress Scale (DASS-21) [18] to assess depressive symptoms and administered it at a baseline laboratory session. Participants rated the degree to which statements applied to them over the past week (eg, “I felt downhearted and blue”) from 0 (“did not apply to me at all”) to 3 (“applied to me very much, or most of the time”). In a way similar to the SIAS analysis, we examined depression on a continuous scale (mean 3.3, SD 2.4). Scores among individuals with a diagnosis of major depressive disorder have been previously documented (mean 15.0, SD 4.6) [18]. Internal consistency of the DASS-21 depression subscale in this study’s sample was excellent (alpha=.91).

#### State Affect (In Situ)

**Figure 1:**
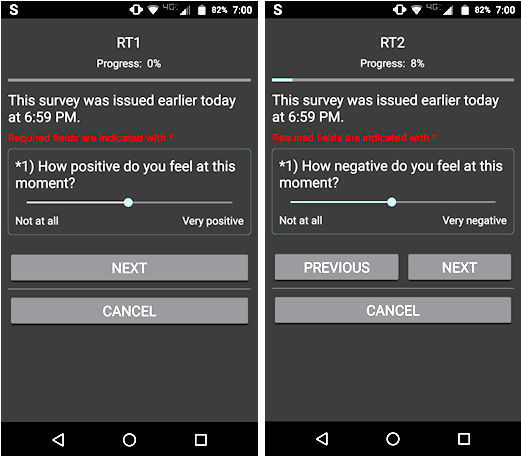
Screenshot of positive (left) and negative (right) state affect rating as seen on a mobile phone screen.

Each day, participants provided up to 6 separate ratings for current positive (“How positive are you feeling?) and negative (“How negative are you feeling?”) affect using a visual analog scale (designed to minimize influence of one’s previous ratings on the current rating) from “not at all” to “very positive” or “very negative.” Each scale was coded from 0 to 100. Sliding scales were always initially presented as at the midpoint to reduce biased reporting (Figure 1).

### Feature Extraction Using Unobtrusive Data

We indexed social isolation by the percentage of time a participant spent at home relative to other locations. While time at home does not necessarily indicate lack of social interaction, it does suggest lack of engagement with external reinforcements, and variants of homestay have been examined in previous research leveraging GPS data [13]. Identification of home location was based on the most frequent appearance of localities of each participant during nighttime (12:00 AM to 6:00 AM) every day, a method used in past research [13]. No participants had multiple home locations. On any given day, GPS data were over 90% (>173/192) complete between the hours of 10:00 AM and 6:00 PM, based on dividing the amount of data collected by the total amount of possible data. We attributed missing GPS data to users turning off their phone or shutting down the app. To handle gaps in location data, we used the last observation carried forward method.

### Procedure

Participants were told that the study examined thoughts and feelings as people interacted with their daily environment, and were allowed to participate in the study if they owned an Android device with operating system 4.3 or higher. All data were collected in the spring semester and participants started the study on a rolling basis. There were no active participants during long scheduled breaks (eg, spring break). Participants completed 2 laboratory sessions separated by roughly 2 weeks (mean 16.41 days, SD 2.69 days). At the first laboratory session, after providing informed consent, participants completed measures of social anxiety and depression. At the end of the laboratory session, research staff assisted participants in downloading the app (Sensus [19]), a mobile crowd-sensing system, onto their personal mobile phones. The app was programmed to prompt participants up to 6 times per day between 9:00 AM and 9:00 PM, occurring at random times within six 2-hour windows; thus, prompts could occur between 2 minutes and nearly 4 hours apart. Due to software bugs, phone compatibility issues, and participants shutting down the app, on average, only 2.5 prompts per day were received. In addition to delivering self-report prompts, the app collected GPS location data continuously over the course of the study. Participants were made aware of the type of data the app would collect from their mobile phones. The app was active throughout the study period until participants came back for the second laboratory session, when they were fully debriefed. Other measures assessing psychosocial factors not directly related to this research were administered at 2 laboratory sessions, as were additional questions during the ecological momentary assessment portion of the study. A full list of measures and questions can be obtained by contacting the lead author (PIC).

### Data Preprocessing

**Figure 2:**
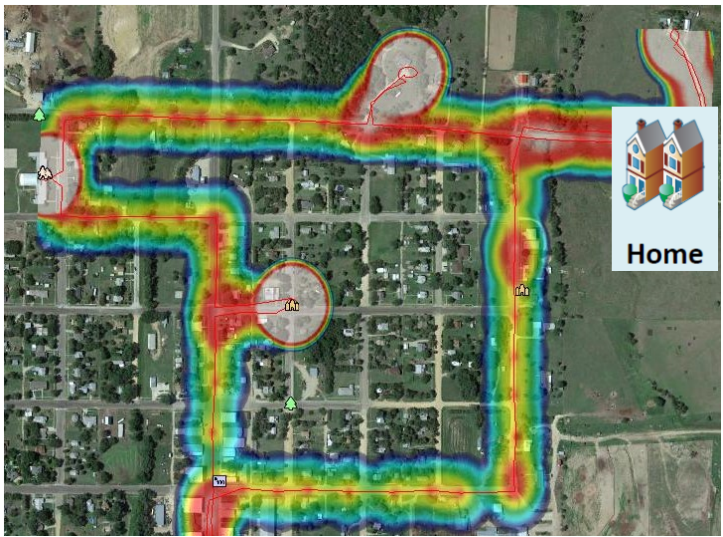
Example of global positioning system (GPS) location data overlaid on a satellite image. The colors indicate the amount of time spent at various locations (more red indicating more time spent at a particular location, with the red line indicating a path connecting various locations).

GPS location information (Figure 2) was collected every 150 seconds and uploaded to a secure Amazon Simple Storage Service server (Amazon S3; Amazon Web Services, Inc, Seattle, WA, USA). Raw GPS data were first clustered according to spatial and temporal locality [20,21]. For this study, we used 30 minutes as the temporal threshold and 30 meters as the spatial threshold. We removed GPS points where the participant stayed less than 5 minutes (eg, roads used for travel) [12,20].

### Data Analysis

In all statistical models, state affect served as the independent variable and time spent at home was the dependent variable. Social anxiety and depression symptoms (using the SIAS and the depression subscale of the DASS-21) were entered as the moderators. We examined 4 different models. The first model examined associations between change in state affect (we examined positive and negative affect separately) across a time window lasting up to 4 hours and time spent at home during that same time window. Change in state affect was computed as the difference between self-reported affect from the start to the end of a time window (computed separately for positive and negative affect). We based the decision to use a window length of up to 4 hours on any 2 random time prompts being timed to go off at a maximum of 4 hours from one another (note that only about 12%, 329/2741, of the total random time surveys were rendered unanalyzable because they were too far apart in timing from another survey). The next 3 models examined the associations between mean-level positive and negative state affect and ratio of time spent at home over the course of a typical workday (10:00 AM to 6:00 PM). This was done for models in which state affect was associated with (1) time spent at home the *same* day, (2) time spent at home the *following* day, and (3) time spent at home the *previous* day (models testing prior day homestay have a predictor that follows the outcome, although we wish to stress that all models tested are correlational).

Due to skewed distribution of the time-spent-at-home variable, we computed 2 sets of analyses using generalized mixed-effects models. In 1 set of analyses, using mixed-effects regression, we entered time spent at home as a continuous variable. Time spent at home scores were log transformed to address right skew. For these analyses, we computed time spent at home as a ratio of the percentage of time an individual spent at home during a set window (ie, within a 4-hour period or over the course of a day), relative to the *average* percentage of time that individual spent at home over the *entire* study period. Thus, the ratio provides an indicator of whether an individual spent more or less time at home over a predefined window relative to the amount of time they typically spent at home during the study. Another set of analyses, using mixed-effects logistic regression, examined the *likelihood* that an individual was at home at some point during that window (in this case, we treated time spent at home as binary, where 1=spent some time at home during predetermined window, and 0=did not spend any time at home during that window).

We conducted all analyses using generalized mixed-effects models and fitted them using the lme4 package in R 3.3.2 (R Foundation) [22]. We computed effect sizes representing the amount of variance explained by the fixed effects in our generalized mixed-effects models using the MuMIn package in R [23]. For analyses with mixed-effects logistic regression, in which time spent at home was a dichotomous variable, we report the unstandardized betas from our models [24]. We used generalized mixed-effects models because they can account for changes over time and missing data more effectively than repeated-measures analyses of variance. In all models, we entered subject and day as separate random intercepts to account for differences in mean responses between participants and between days. For analyses examining time windows within a day, to control for differences in the length of time windows, we entered time window length as a random slope in the analyses.

An example of the model with time spent at home as the criterion variable, state negative affect and depression as predictor variables, and subject as the random intercept is the equation *T*_si_ = *β*_0_ + *β*_1_(*NA*) + *β*_2_(*Dep*) + *β*_3_(*NA* × *Dep*) + *S*_0s_ + *e*_si_, where *S*_0s_~ *N* (0, *τ*^2^_00_) and *e*_si_~ *N* (0, *σ*^2^), and where *T* is time spent at home, *NA* is state negative affect, *Dep* is trait depression, and *S*_0s_ is the random intercept.

Due to the amount of time spent at home during a given time window being evaluated relative to each participants’ personal average time spent at home (ie, a within-subject ratio) for the continuous measure, and the difficulties with interpreting prediction of prior versus same versus next day affect when the predictor was only measured at baseline, we computed a separate set of between-subjects analyses to examine the main effects of depression and social anxiety predicting both time spent at home (without accounting for each participants’ personal average time spent at home) and likelihood of time spent at home. This was done using mixed-effects regression models in which time spent at home (as a continuous or dichotomous variable) was the response variable, depression and social anxiety were predictor variables, and subject and day were random intercepts. We computed separate models for 4-hour time windows and between 10:00 AM and 6:00 PM.

## Results

### Main Effects of Depression and Social Anxiety on Time Spent at Home

As expected, when examining time spent at home using 4-hour windows, there was a significant main effect for social anxiety (standardized beta=.05, *P*=.007), such that a higher level of social anxiety was associated with spending more time at home during 4-hour time windows. Depression was not associated with spending more time at home during 4-hour time windows (standardized beta=.02, *P*=.20). When examining amount of time spent at home between the hours of 10:00 AM and 6:00 PM, as expected, there once again was a significant main effect for social anxiety (standardized beta=.05, *P*=.02), such that a higher level of social anxiety was associated with spending more time at home between 10:00 AM and 6:00 PM. Similarly, those higher in depression tended to spend more time at home between 10:00 AM and 6:00 PM, although this main effect did not quite reach significance (standardized beta=.03, *P*=.06).

When examining the likelihood of time spent at home using 4-hour time windows, as expected, there was a significant main effect for social anxiety (standardized beta=.37, *P*=.02), such that a higher level of social anxiety was associated with a greater likelihood of spending time at home during 4-hour time windows. The main effect of depression (standardized beta=.14, *P*=.35) on likelihood of time spent at home was not significant. When examining time spent at home between the hours of 10:00 AM and 6:00 PM, the main effect of social anxiety was not significant (standardized beta=–.13, *P*=.61). Unexpectedly, there was a significant main effect for depression (standardized beta=–.60, *P*=.001), such that higher depression was associated with a lower likelihood of being at home.

### Associations Between Change in Affect and Time Spent at Home in the Same Day Using Short Windows

We then examined the associations between changes in positive and negative affect and time spent at home within the same (up to 4-hour) time windows. For analyses in which time spent at home was a continuous variable, as expected, there was a significant main effect for change in negative affect (*F*_1,230_=8.61, *P*=.004, *R*^2^=.03), such that a greater increase in negative affect (between the start and end of the time window) was associated with more time spent at home during that window. Similarly, there was a significant main effect for change in positive affect (*F*_1,119_=9.71, *P*=.002, *R*^2^=.03), such that a greater increase in positive affect was associated with less time spent at home during that window. There were no significant interactions between state affect and either depression or social anxiety (all *P*>.10).

For analyses in which time spent at home was a dichotomous variable, there were no significant main effects for positive affect, negative affect, or interactions between state affect and either depression or social anxiety (all *P*>.10).

### Associations Between Affect and Time Spent at Home Within the Same Day

We then examined associations between mean-level positive and negative affect and time spent at home the same day. For analyses in which time spent at home was a continuous variable, as expected, there was a significant main effect for negative affect (*F*_1,105_=5.52, *P*=.02, *R*^2^=.02), such that higher negative affect was related to more time spent at home on the same day. There was no significant main effect for positive affect, or interactions between state affect and either depression or social anxiety (all *P*>.10).

For analyses in which time spent at home was a dichotomous variable, in line with our hypotheses, there was a significant main effect for positive affect (unstandardized beta=–.03, *P*=.01), such that higher positive affect was associated with a lower likelihood of being at home. There was no significant main effect for negative affect, or interactions between state affect and either depression or social anxiety (all *P*>.10).

### Associations Between Affect and Time Spent at Home the Previous Day

For models in which time spent at home was a continuous variable, there was a significant expected main effect for average positive affect (*F*_1,163_=5.16, *P*=.02, *R*^2^=.03), such that greater positive affect was associated with less time spent at home the day before. There was no significant main effect for negative affect, or significant interactions between state affect and either depression or social anxiety (all *P*>.10).

For analyses in which time spent at home was a dichotomous variable, for those higher in depression, a lower level of positive affect was associated with a higher likelihood of time spent at home the previous day, although this interaction did not reach significance (unstandardized beta=–.03, *P*=.09). There were no significant main effects for state affect, or interactions between state affect and social anxiety (all *P*>.10).

### Associations Between Affect and Time Spent at Home the Following Day

For analyses in which time spent at home was a continuous variable, counter to hypotheses, there was a significant main effect for average positive affect (*F*_1,152_=7.02, *P*=.009, *R*^2^=.03), such that higher positive affect was associated with more time spent at home the next day. There was no significant main effect for negative affect, or interactions between state affect and either depression or social anxiety (all *P*>.10).

**Figure 3:**
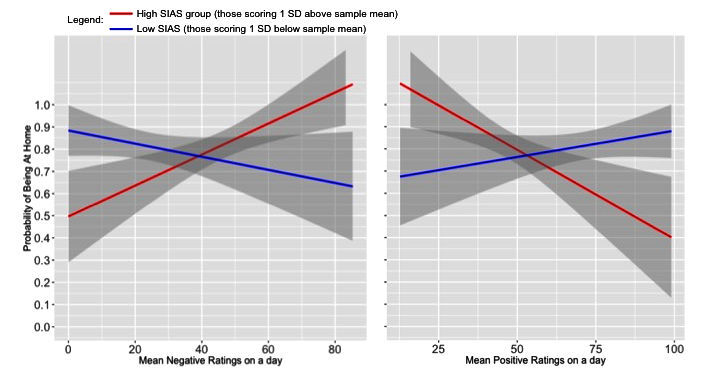
Interactions of mean-level negative (left panel) and positive (right panel) affect with probability of being at home the next day, for those high (1 SD above the mean, in red) and low (1 SD below the mean, in blue) in social anxiety. The Social Interaction Anxiety Scale (SIAS) score was entered as a continuous variable in all models, although to illustrate the interaction effects, only the effects of those high and low in SIAS are plotted.

For analyses in which time spent at home was a dichotomous variable, there were no significant main effects for negative affect or positive affect (*P*>.10). Notably, there was a series of interactions in line with the general hypothesis. Specifically, a significant interaction between state negative affect and social anxiety being associated with time spent at home the next day (unstandardized beta=.05, *P*=.001) indicated that, among individuals with higher social anxiety, greater negative affect over the course of a day was associated with a greater likelihood of spending time at home the next day (Figure 3). The finding was similar for those higher in depression with a high level of negative affect, although this interaction did not reach significance (unstandardized beta=.02, *P*=.07). Further, there was a significant interaction between positive affect and social anxiety (unstandardized beta=–.04, *P*=.007), such that among individuals with greater social anxiety, lower mean-level positive affect over the course of a day was associated with a greater likelihood of spending time at home the next day.

Table 1 shows all of the results for analyses involving the association between changes in affect and time spent at home during 4-hour windows, moderated by depression and social anxiety. It also shows results for analyses involving mean affect and time spent at home on (1) the same day, (2) the previous day, and (3) the following day, moderated by depression and social anxiety, for both the amount of time spent at home and the likelihood of time spent at home. Because of the number of statistical models examined, we conducted a binomial test to determine whether the number of significant effects occurred due to random chance. We chose a binomial test because traditional alpha corrections for type I error rates (eg, Bonferroni) tend to be too conservative, reduce statistical power, and increase the risk of type II errors [25]. Assuming a 5% chance of a type I error for each possible effect in a model (effects were counted as either significant or not), the probability that the 8 (out of a possible 48) significant effects occurred due to chance is <0.001.

**Table 1:** Main effects of state affect, as well as interactions between state affect and either social anxiety or depression, in relation to the amount and the likelihood of time spent at home.

Affect or interactions		Change in affect in 4-hour window		Mean affect the same day		Mean affect the previous day		Mean affect the following day	
		Beta	*P* value	Beta	*P* value	Beta	*P* value	Beta	*P* value
**Standardized betas for amount of time spent at home (as a continuous variable)**									
	NA^a^	.09	<.01	.08	.02	.03	.39	–.05	.12
	NA × SIAS^b^	–.001	.98	–.03	.31	–.05	.19	–.03	.37
	NA × DASS^c^	–.01	.81	–.40	.19	–.56	.11	–.01	.85
	PA^d^	–.09	<.01	.05	.17	–.09	.02	.09	<.01
	PA × SIAS	.01	.66	.04	.23	.03	.40	.004	.90
	PA × DASS	.01	.85	.03	.27	.05	.20	–.01	.67
**Unstandardized betas for likelihood of time spent at home (as a dichotomous variable)**									
	NA	–.02	.71	.01	.24	–.02	.27	–.01	.48
	NA × SIAS	.06	.15	–.01	.29	.01	.51	.05	<.01
	NA × DASS	.06	.25	–.01	.42	.01	.68	.02	.07
	PA	–.03	.49	–.03	.01	–.02	.37	–.01	.49
	PA × SIAS	–.03	.47	–.01	.30	–.02	.22	–.04	<.01
	PA × DASS	–.02	.76	–.001	.92	–.03	.09	–.01	.28

^a^NA: negative affect.

^b^SIAS: Social Interaction Anxiety Scale scores.

^c^DASS: Depression, Anxiety and Stress Scale-depression subscale.

^d^PA: positive affect.

## Discussion

### Principal Findings

This study indicates that it is possible to integrate GPS data, a commonly available source of data from mobile phones, with repeated in situ sampling of (positive and negative) affect to enhance understanding of the relationship between affect and time spent at home, and its interaction with depression and social anxiety. By focusing on just one location-based metric, time spent at home, we were able to explore multiple models that differed in terms of their time windows and temporal links. Consistent with existing theory and past research on affect and social isolation, there were significant main effects for social anxiety and state positive or negative affect across many models, suggesting that there are multiple ways of modeling temporally rich links between affect and homestay depending on the research question. For example, researchers interested in studying the relationship between *changes* in positive affect and social isolation may wish to examine shorter time windows within a day.

Existing work in psychology examining the relations between affect and social isolation has mostly relied on data drawn from self-report measures that can have limited ecological validity. Thus, despite sound theoretical reasons to expect affect to predict social isolation, to date, to our knowledge, virtually no research has investigated how this theoretical relationship is expressed in situ in people’s actual lives. A major contribution of our research is finding that temporal relationships between affect and time spent at home, a marker suggestive of social isolation, can be modeled using fine-grained data from people’s daily lives, through a combination of ecological momentary assessment and passive sensing of location. Thus, research that leverages mobile sensors has the potential to not only test, but also improve, theoretical models from psychology. For example, these methods enable novel tests of the impact of contextual variables on the links between affect and social isolation. It is likely that the degree to which (trait or state) affect is linked to the amount of time someone spends at home (and thus, social isolation), and vice versa, depends on a host of important factors, such as how far someone is from home, who they are surrounded by, biological states such as hunger and fatigue, and even whether data are collected on a weekend or a weekday [26]. As precision of context-aware sensing capabilities continues to be developed, we expect researchers to develop increasingly complex models of behavior that can better account for the richness and variety of everyday life.

We found many of the anticipated main effects of state and trait affect, in that the observed significant effects were almost all in the expected direction (although there were also null findings). There were fewer significant interactions between state affect and symptoms of social anxiety and depression. Interestingly, the reliable interaction findings were with higher negative and lower positive state affect interacting with higher social anxiety, being associated with greater likelihood of spending time at home the following day, suggesting that perhaps the state and trait affect act as a “double whammy.” Specifically, these findings are consistent with a wealth of research demonstrating that, in general, how (good or bad) people feel influences their desire to seek out or avoid others or engage in activities outside the home (in our case, socially anxious individuals tending to spend more time at home). We further found that spending more time at home is associated with higher negative affect that day. Not only that, it may be that for socially anxious individuals, a high level of distress or low level of pleasure on a day is associated with retreating to social isolation the next day, creating a double whammy effect. This makes sense considering that individuals high in anxiety have difficulty disengaging their attention from negative stimuli [27], and it may be hard for socially anxious people to stop thinking about the culmination of distressing (or lack of pleasurable) experiences that occurred the previous day, making them want to isolate themselves from others. Future research may wish to examine this possibility more closely by frequent in situ sampling of both emotions and thoughts, as well as passive sensing of social interaction through a wireless technology such as Bluetooth.

There were some null findings, as well as two significant findings, that were contrary to our expectations. In particular, higher positive affect was associated with more time spent at home the next day, and higher depression was associated with a lower likelihood of being at home between 10:00 AM and 6:00 PM. Rather than trying to come up with post hoc explanations for these findings, we wish to emphasize that this research is preliminary and involved running multiple sets of analyses (because we were interested in looking across different methods of operationalizing the homestay-affect link), which inevitably increased the chance for spurious findings. Thus, although we did not observe some expected main effects and interactions, the overall pattern of findings suggests a relationship between feeling worse (ie, being high in social anxiety, or having high state negative affect or low positive affect) and spending time at home, although replication is needed in larger samples.

### Limitations

These results should be interpreted in light of several limitations. Although we tested different models of affect predicting time spent at home, the data are correlational and no causal relationships should be inferred. Future research that wishes to make causal claims may want to manipulate affect in a subset of participants to determine effects on social isolation and to manipulate time spent at home to observe changes in affect. Further, although we assessed depression and social anxiety using well-validated self-report measures, we did not use a diagnosed sample or administer structured clinical interviews. It is also possible that other factors outside of depression and social anxiety influenced our findings, such as alcohol use, which is commonly used as a way to decrease depressive and anxious symptoms. Although we sought to examine links between trait and state affect, the measures we used do not directly align with one another (eg, depressive symptoms are not synonymous with negative affect). Future research should try to replicate our findings using a community sample, given that undergraduate students may have similar daily routines (eg, due to common class schedules) that influence time spent at home. Given the limitations of statistical power, we were unable to examine the potential impact of sex and race, two factors that should be more closely examined in future research involving larger samples.

In addition, while we used ecological momentary assessment to repeatedly sample affect throughout the study period, we did not obtain physiological correlates of affect such as heart rate and skin conductance. One advantage to examining physiological markers of affect is the ability to unobtrusively and continuously monitor fluctuations in states [28]. It will also be important to assess variability across different metrics of isolation, given that time spent at home is only one metric that can be used to approximate social engagement. For example, it may be possible to obtain a more reliable and precise measure of social isolation by combining time spent at home with Bluetooth sensing of individuals nearby or voice recognition software. This would allow researchers to obtain a more fine-grained understanding of whether and how long social isolation occurs at home. Relatedly, asking users to report on their activities at a given location, or inferring activities from other passively sensed data, would allow for enhanced understanding of how people spend their time at various locations and how this might influence emotions and depressive or anxious symptoms, although it would add a considerable measurement burden. Although we based our inference of home location on previous research, future work may wish to query participants about their work hours (eg, if they work a night shift) or activities between 12:00 AM and 6:00 AM, as these factors may influence ability to infer home location. Furthermore, we experienced some technical difficulties such as software bugs and compatibility issues that prevented us from collecting as much data as we would have liked. However, because these problems occurred at random, we still expect that our data can be used to make inferences about people’s everyday experiences.

### Conclusions

Even taking into consideration these limitations, the ability to integrate fine-grained location data with self-reported affect in situ has tremendous potential to help explicate how short-term, real-time emotional experiences are related to important behavioral patterns in both healthy emotional functioning and in depression and social anxiety. Improving our ability to assess and model variations in affect and GPS patterns may enhance detection of mental disorders through early recognition of signature patterns or change in patterns indicating an increase in isolation, as well as inform treatment planning and assessment of outcomes. Given the ubiquity of mobile phones in our society, understanding how to leverage and integrate seemingly disparate forms of actively and passively sensed data has strong potential to address the growing needs for mental health monitoring and treatment.

## Abbreviations

DASS-21: Depression, Anxiety and Stress Scale
GPS: global positioning system
SIAS: Social Interaction Anxiety Scale
